# Chicago Medical Society

**Published:** 1886-07

**Authors:** 


### SOCIETY REPORTS.

---

CHICAGO MEDICAL SOCIETY.

*Stated Meeting, May 3, 1886.*—THE PRESIDENT, E. J. DOERING, M. D., in the chair.

DR. M. P. KOSSAKOWSKI exhibited a case of

DOUBLE HARE-LIP AND CLEFT PALATE IN AN INFANT.

He promised a subsequent report of the case.

DR. H. GRADLE read a paper on

PURE DRINKING WATER.

He said the only way of deciding the question whether the Chicago drinking water is ever deleterious to health is to search for the number and kinds of micro-organisms it contains. The sewage is poured into Lake Michigan, the source of water supply, whenever the current in the river drifts toward the lake. It is well known that the discharges of typhoid fever—of which there are over 5,000 cases annually in the city—contain the typhoid bacilli. Any spores existing in them, and thus carried into the lake, are likely to remain alive in the water long enough for some few of them to enter the water mains. A similar danger is not unlikely in the case of other intestinal diseases, although the parasites causing them have not yet been identified. Such considerations lead one to suspect the water as the possible cause of infection, however rare.

The speaker has made some culture analyses according to the methods of Koch, and found from 1,000 to 1,500 living germs per cubic centimeter of water. Four to six varieties of bacilli are commonly met with in the water, mostly in the form of spores.

For purification the speaker recommended twenty minutes' boiling, or thorough filtration. Most filters in the market are inefficient. The Mallie aerifilter, however, was found to be practically bacteria-tight, when recently cleansed, though on continued use a few germs passed through it. It consists of a cylinder of porous clay screwed to the faucet, and surrounded by a glass cup, through which the water passes.

PROFESSOR LONG asked if it is demonstrated whether the bacteria in our drinking water comes from the sewerage or from the air.

DR. L. CURTIS said there is no doubt that water absorbs a great number of bacteria from the air, and that these may gain entrance into the circulation through the digestive tract as well as through the respiratory tract. He said there is no doubt of the possibility of malarial germs being introduced into the system by means of drinking water.

DR. R. TILLEY wished to know if it has been actually demonstrated that the bacillus tuberculosis finds its way through our sewers into the lake and drinking water. We are not justified in saying that our water contains this element of danger until it has been demonstrated.

DR. J. ZEISLER asked if Dr. Gradle had examined the Waukesha waters and found bacteria in them.

DR. GRADLE closed the discussion by saying he took pains not to state in his paper whether he thought the disease germs found in the water were introduced by means of the sewerage or air. It is not a vital point so far as the use of the water is concerned, although if it is decided that these bacteria are introduced by means of the sewerage and not the atmosphere, it would have a bearing on the question of where to dispose of our sewerage. However, there is no doubt but bacteria are introduced into large bodies of water by means of dust, vegetation, and decayed leaves, and there is no reason to believe that the bacteria found in our water are derived from the sewerage alone. A Russian chemist had examined the water found at St. Petersburg, and found that at a distance from the city the number of bacteria per cubic centimetre was considerably less than near the city. Dr. Gradle had examined water obtained from Lake Michigan about thirty-five miles from Chicago and had found about the same number of bacteria per cubic centimetre, but a less number of varieties than in city water. He believed the spores of typhoid fever and tuberculosis may find entrance into our lake water and become causes of disease in individuals who are not prepared to resist their attack. Dr. Gradle had never examined the Waukesha waters, but he had examined water from a spring on his own farm, which he believed to be as pure as Waukesha water, and had found only fifteen to twenty bacteria per drop, and only three varieties. He believed that spring waters are the purest, as the bacteria they contain cannot be due to sewage.

DR. W. H. LYFORD read a report of a case of

### SCLERODERMA.

A girl, æt. 10 years, apparently healthy and of good family history. Five years ago she received a shock from a lightning stroke. Shortly afterward her parents noticed, under the surface of the skin over the outer region of the left forearm, a delicately traced symmetrical figure, branching and ramifying in purplish colored lines with singular regularity. Dr. Lyford believed the lightning had paralyzed the cutaneous branches of the musculo-spiral nerve, and caused congestion of these filaments, with the marking as a result. In due course of time the skin has undergone the changes that take place in scleroderma. There is hyperæsthesia accompanied by pain, especially at night. The patch is also now slightly elevated above the surrounding healthy integument, and is only slightly movable, while at the elbow it is attached to the fascia and sheaths on the tendons, thus interfering somewhat with the movements of the forearm.

DR. J. ZEISLER said he had seen four cases of scleroderma, two of general scleroderma and two of partial scleroderma.

The first two cases he saw in one of Kaposi's clinics. Their appearance was striking. In scleroderma the features of the face are immobile, and therefore the patient cannot express the emotions. The skin and underlying tissues seem to grow together. The cases of partial scleroderma he had seen in this city. In one patient the patch of scleroderma was a ribbon-like band behind one ear, pinkish in color, immovable upon the underlying tissues. The other case was a lady æt. 50 years, who noticed her right mammary gland was becoming hard, large, and the nipple contracted. There was no pain in the breast nor were the axillary glands involved. The skin was hard and adherent to underlying tissue. A similar condition was observed in a patch of skin on the right arm. Kaposi states the majority of cases of scleroderma occur in females. The best treatment for scleroderma is massage and galvanic electricity.

DR. H. GRADLE presented some stereoscopic views of sections of hypertrophied tonsils taken from Troutman's book on "Hypertrophied Tonsils," after which the Society adjourned.

---

CHICAGO MEDICAL SOCIETY.

*Stated Meeting, May 17th, 1886.*—THE PRESIDENT, E. J. DOERING, M. D., IN THE CHAIR.

PROFESSOR EDMUND ANDREWS exhibited a

NEW EVACUATOR FOR LITHOLOPAXY, and said that one serious defect of Bigelow's and Thompson's evacuators is that the rubber bulb makes suction only for an instant instead of continuously, thus allowing fragments of stone which lie along the tube to be thrown back into the bladder when the bulb is compressed, thus irritating the bladder as well as causing it to be repeatedly expanded. To obviate these difficulties Professor Andrews has had constructed an evacuator in which the essential features consist in a double-chamber evacuating tube, straight or curved, the upper chamber being composed of thin metal 8½ millimetres in diameter, terminating in a round tip with fenestrum for evacuating, the under chamber being semi-cylindrical, fitting under the upper chamber so as to make the whole tube oval-shaped, with a diameter of 31½ millimetres. To the under or inflow tube is attached a rubber tube three yards in length, with an inside diameter of one centimetre, to the outer end of which is fitted a metallic strainer large enough to admit without resistance all the water which will flow through the tube. A bucketful of warm carbolized water, 1½ per cent, is hung over the operating table high enough for the surface of the water to be forty-two inches above the pubis of the patient. When it is desired to wash out the bladder the evacuating tube is introduced with the fenestrum towards the patient's head and the tip of the tube pressing towards the rectum, thus making a hollow in the bladder into which the fragments of stone fall. The strainer having previously been introduced into the warm carbolized solution and the rubber tube filled, this tube is attached to the inflow tube and the stopcock turned. The water now enters the inflow tube and passes into the bladder through the small perforations with sufficient force to rapidly drive the fragments of stone through the fenestrum and out of the evacuator. The current is continuous and rapid; in a recent case of litholopaxy where the calculus was hard oxalate of lime an inch in diameter the fragments were washed out in ten seconds. When the inflow tube is closed by a fragment of stone in the fenestrum the operator simply presses on the last four inches of this tube, which is of rubber, and this dislodges the fragment.

Professor Andrews also called attention to a device for securing to any instrument filiform bougies by means of a split screw passing over the bougie secured by a nut, both fitting into the outer end of the catheter.

DR. W. T. BELFIELD opened the discussion by saying that the disadvantages of the ordinary evacuating apparatus mentioned by Dr. Andrews were well recognized, and that the apparatus exhibited seemed adapted to remove them. It possessed, however, one element of danger, namely, the possibility of undue distension of the bladder through sudden clogging of the exit tube. In cases of concentric hypertrophy, where the bladder can contain only three or four ounces, the continuation of the powerful inflow might, if the exit were obstructed, cause serious damage. Of course if the clogging is immediately discovered, a prompt use of the stopcock would prevent injury.

As to the urethral instrument, he would wish to be sure that the filiform bougie could not be detached beyond the stricture. This danger attaches, of course, to all methods of securing the filiform, but would seem to be especially great in the arrangement exhibited. The loss of a filiform in a tight stricture is an extremely uncomfortable accident; in several cases surgeons have even made external urethrotomy to recover it.

To Professor Andrews's remark that in case of such an accident urethrotomy is readily avoided by inserting a small lithotrite into the bladder and seizing the filiform, Dr. Belfield replied that when there is present a stricture so tight as to require patient work to get a filiform to pass, a small lithotrite could hardly enter the bladder unless reinforced by a sledge-hammer. If the urethra is everywhere large enough to admit a small lithotrite, there could be no danger of losing a filiform because there would be no occasion to use one.

DR. FENN wished to know, if the momentum could be increased by increasing the specific gravity of the fluid, what would be the effect of a greater elevation of the reservoir?

Professor Andrews said that the attachment of a flexible guide to the urethrotome is always a source of care lest it become detached. This was less liable to occur with his instrument than in the old way. In case it should happen the lost guide could be seized and drawn out with a lithotrite.

The question of the pressure of the fluids and the strength of the bladder is one of great interest. But little is known about it. No more force should be used on a distended bladder, certainly, than a normal one could readily endure. We get the measure of this to some extent in the pressure of the expulsive power of the organ, which is a safe limit to keep inside of in the absence of other data. The pressure which Bigelow's evacuator ordinarily gives may form another guide, as this is known to be harmless. The reservoir must not be placed so high as to cause dangerous distension, should the outflow tube become obstructed. In practice forty inches had given all the current needed, and this was doubtless a safe pressure to use in any and all cases.

---

DR. JAMES I. TUCKER read a paper on

UNDIAGNOSABLE MALADIES.

These cases generally are not recorded because there is so little about them that is tangible. They are perhaps functional derangements simulating organic diseases. Sometimes these cases yield readily to simple remedies, but are puzzling because so evanescent. At other times they are graver, often ending fatally and yielding no facts upon post-mortem examination which will aid in a correct diagnosis. But the list of undiagnosable ailments is rapidly decreasing ; for example, Richard Bright in 1827 explained that dropsical effusions are frequently due to diseases of the kidneys. Thomas Addison in 1855 ascribed to disease of the suprarenal capsule the cause of a form of anæmia accompanied by a dingy discoloration of the skin. Until recently herpes zoster frontalis was classified among skin diseases, but now with much accuracy it is traceable to disease of Gasser's ganglion. Dr. Tucker related a case in which a lady had been twice badly frightened and her nervous system had been severely prostrated. At intervals she was attacked with an epileptiform seizure, transient paralysis of the entire left half of the body, constriction of the larynx, a state of trance, and finally a trance-like state in which she had died. This patient's mother, after a period of nervous disorder, had become a paraplegic, her father was temporarily insane, the eldest brother has an undefinable nervous disorder and a younger brother spasmodic asthma, while a sister has attacks of recurrent chorea major. In this peculiar group of nervous disorders what is the underlying cause ? It is as yet unknown. This case illustrated the difficulty which hedges about the diagnosis of hundreds of cases of disease.

DR. PEARSON illustrated the difficulties physicians have to overcome in making a diagnosis by reason of the fact that they overlook some points in the history of a case, as in a case in which a patient had swallowed a piece of a metal spoon and it had lodged in the duodenum and blocked up the portal vein.

DR. W. T. BELFIELD presented specimens of

ANCHYLOSOTOMUM DUODENALE.

These had been referred to him for verification by Dr R. W. Gelbach, of Mendota, Ill., who had discovered them in the intestines of young cats that had died of anæmia. Dr. Belfield confirmed Dr. Gelbach's identification of the worms, but for further verification sent them to Professor Joseph Leidy, of Philadelphia, who replied he thought they were anchylostoma, although he had never seen authentic examples. Dr. Belfield said the anchylostoma are small nematode worms, about half an inch long, which inhabit the duodenums of men and cats, and probably of other animals. Discovered in 1838, their pathogenic significance was recognized by Griesinger in 1851, who found in them the cause of Egyptian chlorosis. They are veritable leeches which fasten themselves to the intestinal mucous membrane and suck the blood of their host. When present in large numbers they induce pernicious and fatal anæmia by exhausting the individual's blood. In tropical climates, particularly Egypt and Brazil, cases of pernicious anæmia or chlorosis produced by them are quite frequent ; in Brazil such cases are quickly cured by administering the pulp of fresh figs, which destroys the worms. Quite recently anchylostoma have been searched for and found in patients dead of pernicious anæmia in Germany. So far as Dr. Belfield knew, Dr. Gelbach is the first to discover the worms in the northern States of the Union. Their presence in cats makes it probable that they infest human beings also in this latitude. The possibility of this cause of pernicious anæmia should therefore be kept in mind, especially when other recognized causes—chronic nephritis, malaria, etc.—cannot be detected.

PROFESSOR E. ANDREWS asked if these worms were ever found in sufficient numbers to cause death, and he was answered affirmatively.

PROFESSOR W. W. JAGGARD exhibited the head of the *tænia mediocanellata* obtained in the practice of Dr. C. G. Smith. The following was the formula used:

| | | |
|---|---|---|
| ℞. | Chloroformi ...... ......... ............ | f ʒi. |
| | Oleores. felicis maris.................. | f ʒi. |
| | Ol. tiglii...... ......... .................... | gt. I. |
| | Aquæ camphoræ...... ...... ......... | f ℥ii. |
| | Gum. acaciae q. s. ft. emuls. | |

---

MICHIGAN STATE MEDICAL SOCIETY.

The twenty-first annual meeting of the Michigan State Medical Society was held at Jackson, June 9th and 10th. There was quite a large attendance of representative medical men from all parts of the state. The meeting was called to order by the President, Dr. E. P. Christian, of Wyandotte. Professor Stark, of the Congregational Church, opened the session with prayer. The address of welcome was made by Mayor Bennett. Dr. Main presented the report of the Executive Committee and invited the members of the society to attend a reception to be held at Assembly Hall tomorrow evening. Professor Frothingham presented a report on Ophthalmology. A paper on Foreign Bodies in, and inferior to, the Eyeball, by Dr. Connor, of Detroit, was of much interest. "A proposed plan of Sanitation" was the subject of a paper by Dr. Wight, of Detroit, and a select committee was appointed to present the subject to the next legislature. Professor Maclean gave some recent experiences in surgery, which were interesting and well received. The two first hours of the afternoon session were taken up with papers relating to diseases of females and discussions of the papers. The evening session opened at 8 o'clock. The President read his annual address. Dr. Brown, of Detroit, read a paper entitled "Irritation of the Alimentary Canal." The subject was quite freely discussed. Several motions relative to miscellaneous business were passed. Dr. Ward, of Langsburg, read a humorous poem entitled "Medical Milk." The charges which were brought against Dr. Kellogg, of Battle Creek, for unprofessional conduct and were not sustained by the County Medical Association, were brought before the State Society and were referred to the Judicial Committee. A paper entitled "Some Special Points for Operative Surgery," by Dr. De Camp, of Grand Rapids was well received. Dr. Sullivan, of Ann Arbor, presented a paper entitled "Observations on the Administration of Chloroform." Dr. Herdman, of Ann Arbor, read an interesting paper on "Convenient Manner of Handling Patients Afflicted with Spinal Disease." Dr. Clark presented a paper entitled "Disinfectants and Sanitation." Dr. Long, of the U. S. Army, read a paper on "Small-Pox Inspection Service." A large number of applications for membership were favorably acted on. On motion, five hundred dollars was voted to the International Medical Congress to be held at Washington next season. The next meeting of the society to be held at Lansing, and the time of meeting changed to

Thursday and Friday instead of Wednesday and Thursday, as heretofore. Dr. Ranney, the Secretary, read an interesting report of the society since its organization. The Doctor has been a most efficient secretary for twenty-one years, but owing to the fact that he would spend most of the coming year abroad, he would be compelled to decline a reëlection. The Doctor was made an honorary member. The Nominating Committee reported as follows : *Vice-Presidents*, Dr. Walker, Dr. Hemingway, Dr. Stoddard, Dr. Newkirk ; *Secretary*, Dr. Duffield ; *Treasurer*, Dr. Hagadon ; *Judicial Committee*, Dr. Boise, Dr. Main, Dr. Christian.

